# Magnetic Polyethyleneimine Nanoparticles Fabricated via Ionic Liquid as Bridging Agents for Laccase Immobilization and Its Application in Phenolic Pollutants Removal

**DOI:** 10.3390/molecules27238522

**Published:** 2022-12-03

**Authors:** Runtang Liu, Wei Zhang, Shushu Wang, Huajin Xu, Yi Hu

**Affiliations:** State Key Laboratory of Materials-Oriented Chemical Engineering, School of Pharmaceutical Sciences, Nanjing Tech University, Nanjing 210009, China

**Keywords:** phenolic pollutants, laccase, magnetic polyethyleneimine nanoparticles, ionic liquids

## Abstract

In this study, polyethyleneimine was combined with magnetic Fe_3_O_4_ nanoparticles through the bridging of carboxyl-functionalized ionic liquid, and laccase was loaded onto the carrier by Cu^2+^ chelation to achieve laccase immobilization (MCIL–PEI–Cu–lac). The carrier was characterized by Fourier transform infrared spectroscopy, scanning electron microscope, thermogravimetric analysis, X-ray diffraction analysis, magnetic hysteresis loop and so on. MCIL–PEI–Cu–lac has good immobilization ability; its loading and activity retention could reach 52.19 mg/g and 91.65%, respectively. Compared with free laccase, its thermal stability and storage stability have been significantly improved, as well. After 6 h of storage at 60 °C, 51.45% of the laccase activity could still be retained, and 81.13% of the laccase activity remained after 1 month of storage at 3 °C. In the pollutants removal test, the removal rate of 2,4-dichlorophenol (10 mg/L) by MCIL–PEI–Cu–lac could reach 100% within 10 h, and the removal efficiency could still be maintained 60.21% after repeated use for 8 times. In addition, MCIL–PEI–Cu–lac also has a good removal effect on other phenolic pollutants (such as bisphenol A, phenol, 4-chlorophenol, etc.). Research results indicated that an efficient strategy for laccase immobilization to biodegrade phenolic pollutants was developed.

## 1. Introduction

Phenolic compounds are widely used in the industrial fields of fine chemicals production such as medicines, pesticides, dyes and daily chemicals, while this also leads to a large amount of phenolic pollutants in the wastewater generated in these fields [1,2,3]. These phenolic pollutants cause serious harm to nature and human health [4,5]. How to effectively remove pollutants from wastewater has become one of the key concerns in the environmental field.

The traditional treatment methods for phenolic pollutants mainly include physical adsorption and chemical catalysis [6,7], but these methods usually have the disadvantages of low efficiency and easy to cause secondary pollution. The enzymatic method is considered to be an effective method for degrading phenolic pollutants due to its high specificity, environmental friendliness and high catalytic efficiency [8,9]. Laccase is a kind of oxidoreductase with copper as the catalytic center. It has strong redox ability and a wide range of substrates, and the only by-product is water, which is very friendly to the environment [10,11]. However, free laccase will face a very complex environment when treating actual industrial wastewater, which can easily reduce the activity and stability of laccase [12]. In order to solve these problems, the development of a suitable immobilized enzyme carriers is of great significance to improve the thermal stability, storage stability, catalytic efficiency and reusability of laccase.

Up to now, the materials used to immobilize laccase include nanomaterials, membrane materials, microspheres and so on [13,14,15]. Among them, magnetic nanomaterials have the advantages of easy separation, good mechanical stability, easy surface modification, low toxicity and so on, which have been widely used in laccase immobilization [16,17]. However, individual magnetic material often has the disadvantages of easy agglomeration, low loading and low activity retention, so its surface needs to be modified to improve this phenomenon [18]. In recent years, various surface modification methods of magnetic materials have been developed, which solve these problems to a certain extent. However, it is difficult to greatly improve the immobilized enzyme activity, stability, reusability and other properties at the same time. For example, Ran [19] et al. used polyethyleneimine to directly coat magnetic materials for the immobilization of laccase. Although the loading of laccase reached 70 mg/g, the activity retention was only 80%. Meanwhile, Xia [20] et al. combined amino-functionalized magnetic nanoparticles (Fe_3_O_4_-NH_2_) with polyethyleneimine using glutaraldehyde as a crossing-linker (Fe_3_O_4_-NH_2_-PEI) to immobilize laccase via Cu^2+^ chelation. Although the loading of laccase is only 26.13 mg/g, its activity retention rate reaches 107.41%. The reason for the increased activity may be that Cu^2+^ coordination can reduce the deformation and desorption of laccase, and Cu^2+^ can activate laccase to a certain extent and enhance the activity of laccase [21,22].

Generally, ionic liquids composed of organic cations and anions could be used as green solvents and additives. In our previous study, functionalized ionic liquids were used as novel surface modifiers or bridging agents of organic–inorganic nanomaterials for porcine pancreatic lipase and laccase immobilization. Various enzymatic properties such as the enzyme loading, activity, stability and repeatability of the immobilized enzyme were effectively improved [23,24,25].

**Scheme 1:**
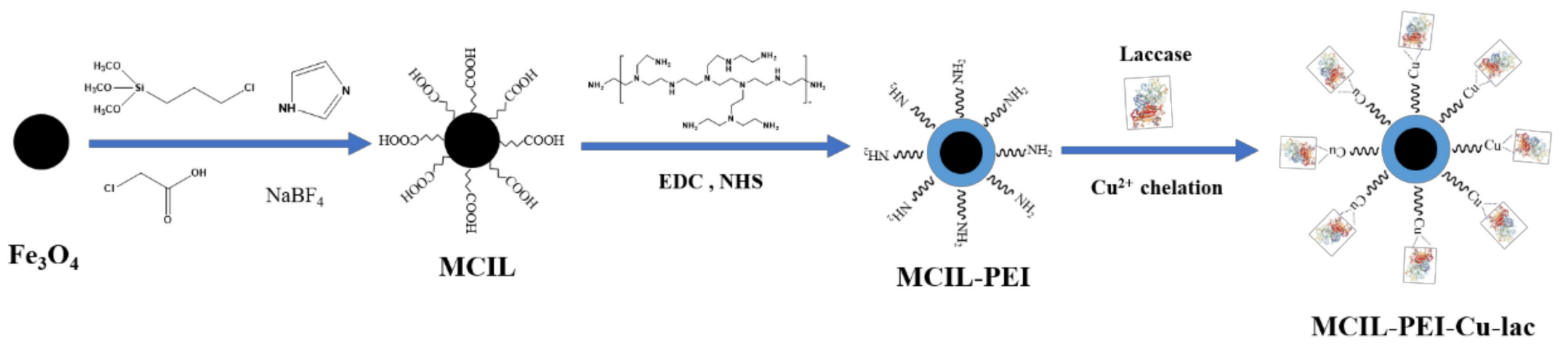
The synthetic route map of magnetic polyethyleneimine nanoparticles.

In this study, carboxyl functionalized ionic liquid was used as a bridging agent to combine magnetic Fe_3_O_4_ nanoparticles with polyethyleneimine, and laccase was immobilized on magnetic polyethyleneimine nanoparticles by copper ion chelation (Scheme 1). Subsequently, the removal ability of immobilized laccase for phenolic pollutants was investigated. We wish to combine the advantages of magnetic nanoparticles, functional ionic liquids, polyethyleneimine and Cu^2+^ activation to prepare an efficient carrier for laccase immobilization. We hope to propose an effective solution to the current bottleneck problem of laccase immobilization.

## 2. Results and Discussions

### 2.1. Analysis of Characterizations

**Figure 1:**
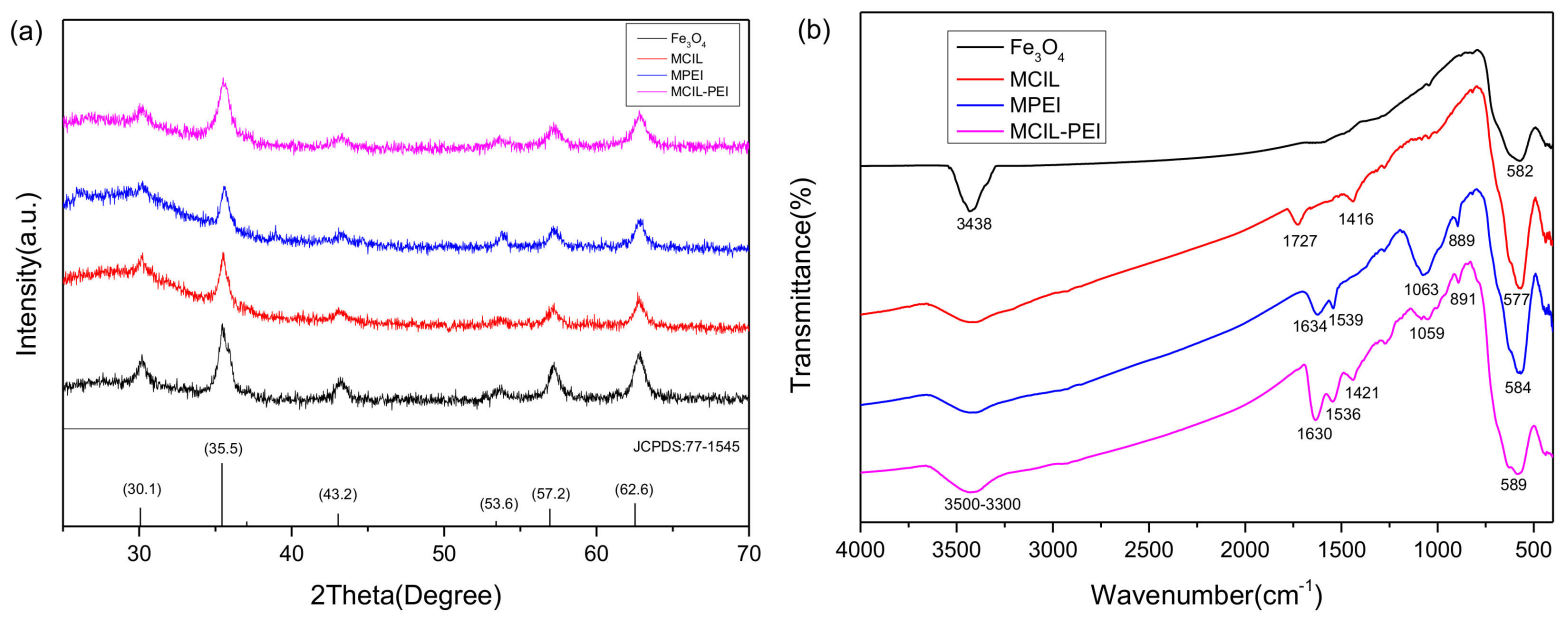
XRD pattern (**a**) and FT-IR spectra (**b**) of carriers.

Figure 1a shows the XRD characterization of Fe_3_O_4_, MPEI, MCIL and MCIL–PEI nanoparticles. The peaks at 2θ = 30.1°, 35.5°, 43.2°, 53.5°, 57.2° and 62.6° are consistent with the characteristic peaks of magnetic nanoparticles Fe_3_O_4_ (JCPDS = 77–1545) reported in the literature [26]. After further modification by carboxyl-functionalized ionic liquid and polyethyleneimine, carries still maintained the same peaks, and the peaks’ intensities were basically unchanged, which indicated that the crystal structure of carries was well preserved.

Figure 1b shows the Fe_3_O_4_, MCIL and MCIL–PEI FT-IR spectrum. In the Fe_3_O_4_ spectrum, the peak at 582 cm^−1^ is the characteristic peak of Fe_3_O_4_, and the peak at 3438 cm^−1^ proves that Fe_3_O_4_ is rich in hydroxyl groups [27]. In the MCIL spectrum, the absorption peak at 1727 cm^−1^ is attributed to the C = O stretching vibration of the carboxyl group, while the new peak at 1416 cm^−1^ is due to the C = C stretching vibration of the imidazole ring in the ionic liquid [28]. These peaks proved that Fe_3_O_4_ was successfully modified by carboxylated ionic liquids. In the MPEI spectrum, the characteristic peaks of polyethyleneimine were successfully detected, which proved that polyethyleneimine was successfully grafted to the surface of Fe_3_O_4_. In the MCIL–PEI spectrum, the broad peaks at 3500–3300 cm^−1^ could be attributed to the simultaneous presence of -NH_2_ and -NH groups in the material [29]. Furthermore, 1630 cm^−1^and 1536 cm^−1^ are the N–H bending vibration peak of primary and secondary amine, and 1059 cm^−1^ is the stretching vibration peak of C–N [30]. At the same time, the peak at 1421 cm^−1^ could be attributed to the C = C stretching vibration of the ionic liquid’s imidazole ring, which does not appear in the MPEI curve. This proves that the MCIL–PEI carrier contains an ionic liquid structure. The absorption peak at 891 cm^−1^ indicates the existence of a tertiary amine structure in MCIL–PEI [19]. The above results indicated that carboxyl-functionalized ionic liquid and PEI were successfully modified onto the surface of MCIL nanomaterials.

**Figure 2:**
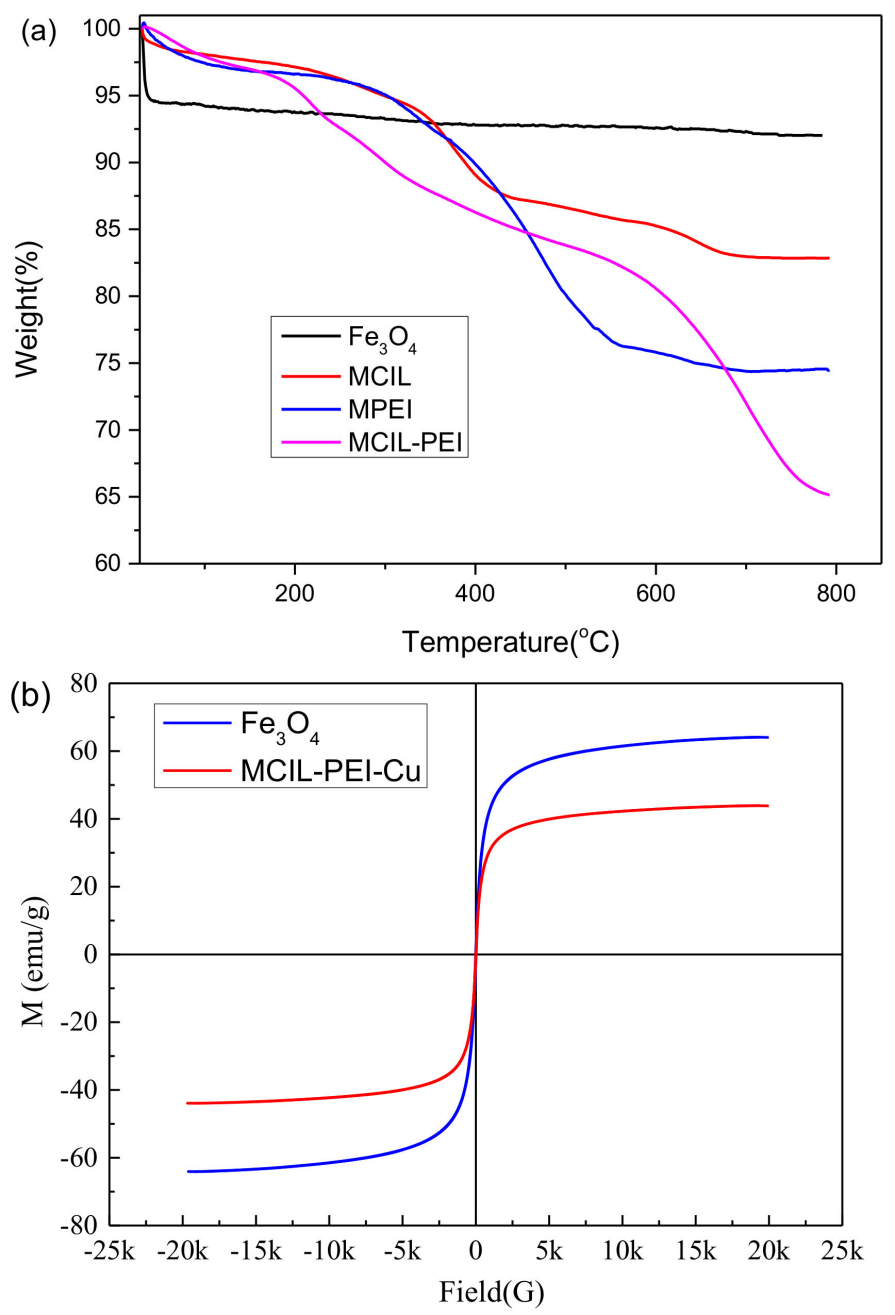
TGA curve (**a**) and magnetic hysteresis loops (**b**) of carriers.

Thermogravimetric analysis of Fe_3_O_4_, MCIL and MCIL–PEI was performed, and Figure 2a shows the TGA curves of the samples. From the TGA curve of Fe_3_O_4_, the weight dropped 7.91%, which may be due to the evaporation of water adsorbed on Fe_3_O_4_. The TGA curve of MCIL dropped significantly after 300 °C, indicating that the grafted carboxylated ionic liquid structure undergoes rapid thermal decomposition, accounting for 11.47% of the total weight. The weight loss of the MPEI TGA curve before 200 °C should be the loss of moisture in the material. After 300 °C, the weight loss speed was accelerated, and finally, 74.47% of the weight remained. It showed that PEI was successfully grafted to the surface of Fe_3_O_4_, and MPEI was successfully synthesized. From the TGA curve of MCIL–PEI, it can be seen that when the temperature reached 200 °C, the decreasing speed of the total weight begins to accelerate. At the same time, when the temperature reached at 610 °C, the structure of the carboxyl-functionalized ionic liquid was completely decomposed. However, the weight was still decreasing at this time, indicating that the polyethyleneimine was not completely decomposed. The final total weight loss indicated that MCIL–PEI contained 18.31% polyethyleneimine. TGA analysis showed that magnetic nanoparticles were successfully modified with carboxylated ionic liquids and polyethyleneimine.

The magnetic hysteresis loops of carriers are exhibited in Figure 2b. Fe_3_O_4_ and MCIL–PEI–Cu nanoparticles exhibit excellent superparamagnetism [27], which is beneficial to the separation and reuse of immobilized laccase. The saturation magnetization of Fe_3_O_4_ and MCIL–PEI–Cu are 64.1 and 43.8 emu/g. The grafting of ionic liquid and polyvinyl imine reduced the magnetization of the carriers.

**Figure 3:**
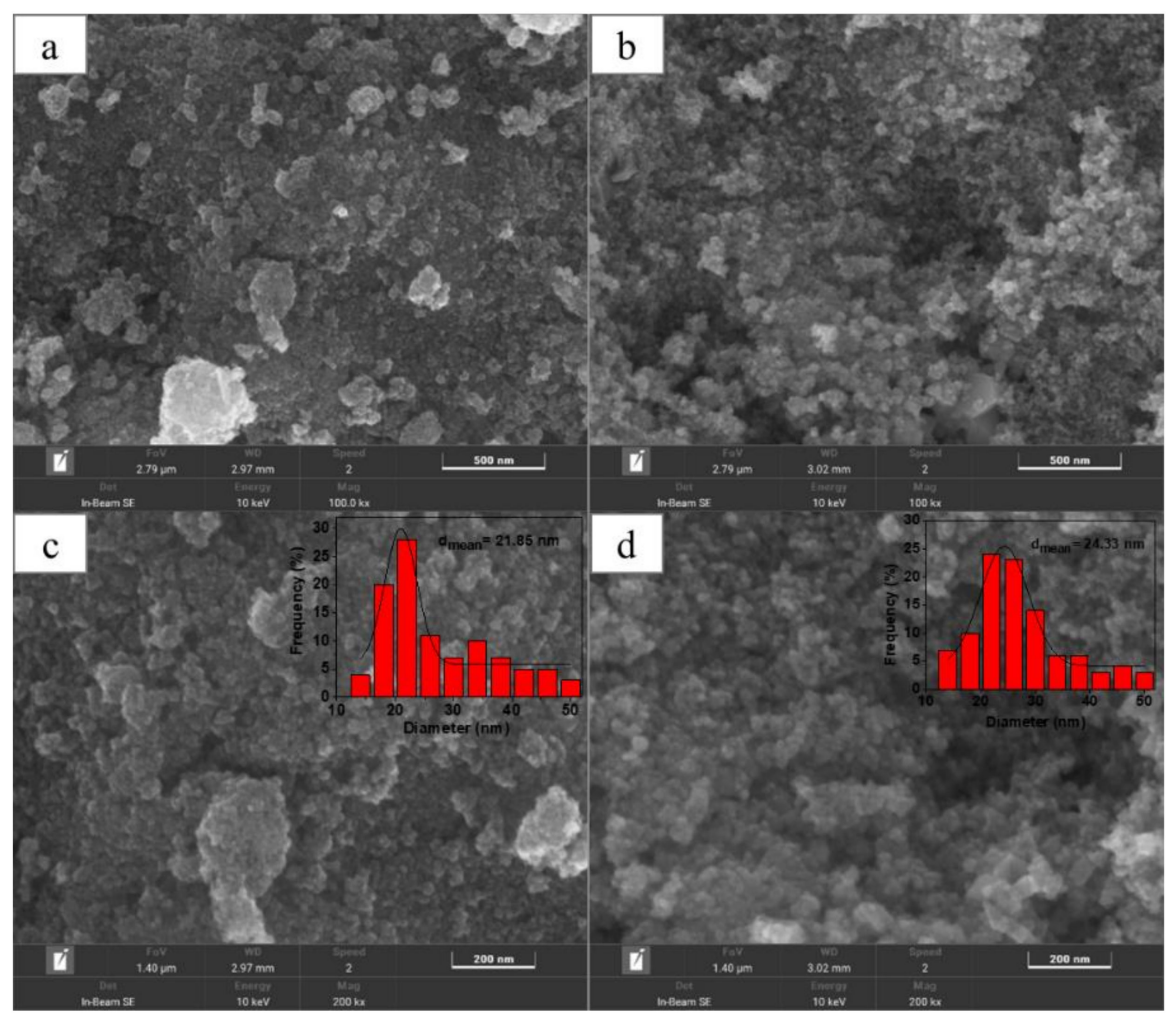
SEM images of Fe_3_O_4_ (**a**,**c**) and MCIL–PEI–Cu (**b**,**d**).

**Figure 4:**
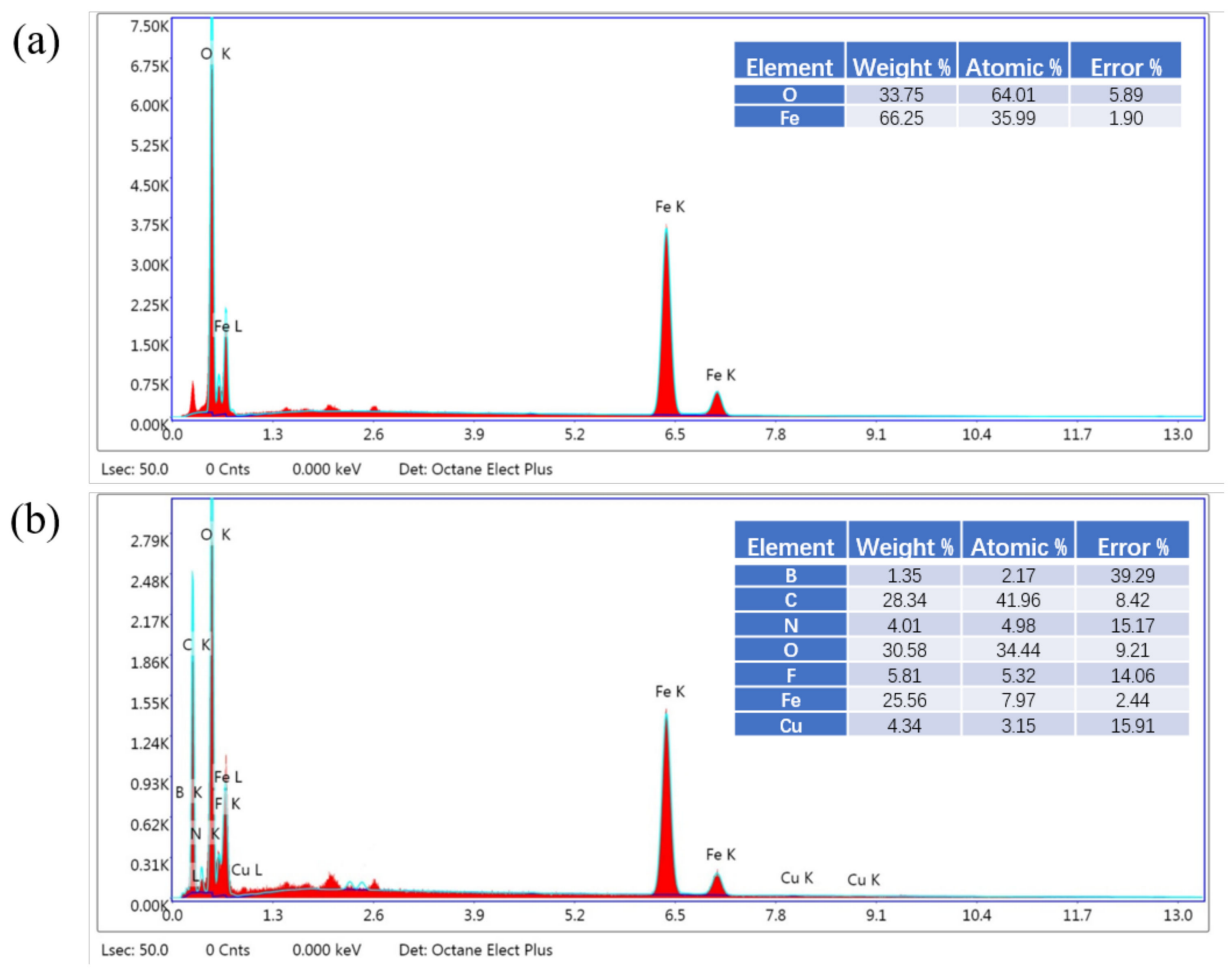
EDS characterization of Fe_3_O_4_ (**a**) and MCIL–PEI–Cu (**b**).

It can be observed from Figure 3 that the prepared Fe_3_O_4_ (a, c) is a nanoscale material with a particle size of 21.85 nm, but the agglomeration phenomenon is very obvious [31]. The modified carrier MCIL–PEI–Cu (b, d) was still spherical or elliptical, but the particle size of MCIL–PEI–Cu is 24.33 nm, which is improved compared with Fe_3_O_4_. At the same time, PEI is a water-soluble cationic polymer with high density of various amino functional groups [22,32]. It can prevent particle agglomeration by using electrostatic repulsion and steric hindrance [33,34]. The dispersibility of MCIL–PEI–Cu nanoparticles is greatly improved compared with Fe_3_O_4_ nanoparticles, indicating that polyethyleneimine has been successfully grafted on the surface of Fe_3_O_4_. From EDS characterization (Figure 4), in the EDS spectrum of MCIL–PEI–Cu, the newly added elements C, N, F and B indicate that the ionic liquid and PEI were successfully modified to the surface of magnetic nanoparticles, and the presence of Cu elements meant the successful introduction of Cu ions.

### 2.2. Results of Laccase Immobilization

As shown in Table 1, compared with MPEI, the loading capacity and activity retention of laccase immobilized on MCIL–PEI were improved to a certain extent. This indicated that the introduction of carboxyl-functionalized ionic liquid groups has a positive effect on the retention of laccase activity while increasing the enzyme loading capacity. The possible reason is that the ionic liquid structure provides a good microenvironment for the immobilization of laccase. Additionally, the introduction of ionic liquid can further enhance the interaction between the enzyme and the carrier, which is conducive to maintaining the integrity and activity of the enzyme conformation. Meanwhile, it has a strong hydrogen bonding ability, which better protects the spatial conformation of laccase. Compared with the above two carriers, MCIL–PEI–Cu has an obvious improvement in enzyme loading and activity retention. Obviously, the introduction of Cu^2+^ increases the action sites of the carrier through coordination, so that the laccase has more binding sites and greatly increases the laccase loading. On the other hand, the introduction of Cu^2+^ can significantly enhance the activity of immobilized laccase, and its activity retention rate is as high as 91.65%, showing a higher level of activity retention than previously reported in literature. Seyed Mehdi [35] et al. used aminated magnetic ferric oxide to immobilize laccase through glutaraldehyde cross-linking, retaining only 27% of its activity. Chen [36] et al. modified the surface of magnetic nanoparticles with polydopamine and immobilized laccase with dialdehyde starch as a cross-linking agent, but the activity retention was only 69%. It might be that Cu^2+^ had a certain activation effect on the active center of laccase, which was consistent with previous literature reports [37].

### 2.3. Stability Test

**Figure 5:**
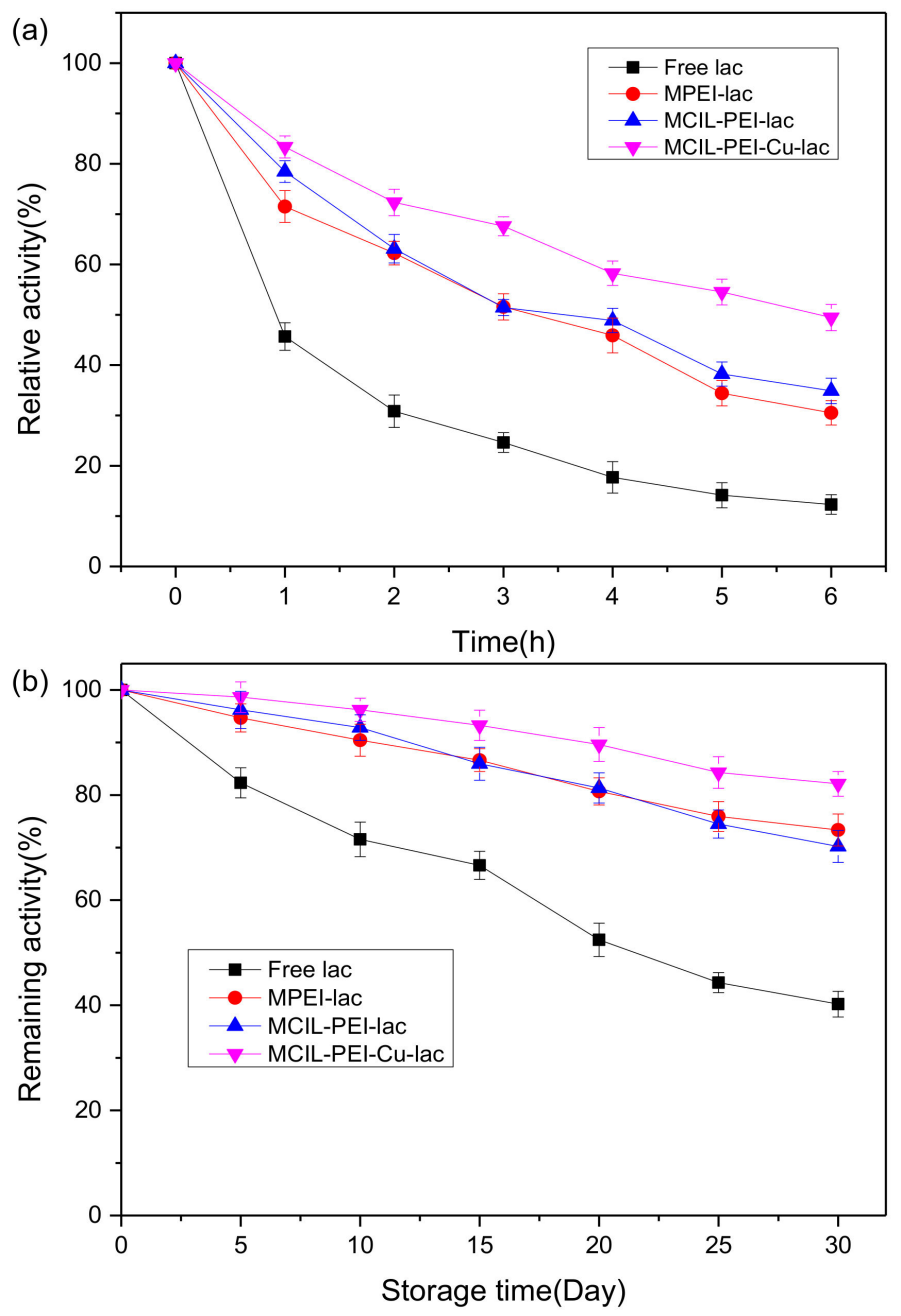
Studies on thermal (**a**) and storage stability (**b**).

A stability test is also necessary to study whether the immobilized laccase has industrial application value. As shown in Figure 5a, the activity of free laccase decreased significantly at a high temperature of 60 °C, and only 12.31% of the enzyme activity remained after 6 h. The thermal stability of three immobilized laccases was greatly improved compared with free laccases. The best immobilized laccase, MCIL–PEI–Cu–lac, still retained 51.45% of the laccase activity after 6 h. In the storage stability test (Figure 5b), the free laccase only retained 40.21% of its enzyme activity after 30 days of storage. While MCIL–PEI–Cu–lac had the highest remaining activity after 30 days of storage, with a remaining activity of 81.13%; it also had a certain degree of improvement compared with other literature reports. Wu [38] et al. immobilized laccase with biomass-derived nanocellulose aerogels. After the immobilized laccase was stored at 4 °C for 20 days, the laccase activity remained at 66.4%. Taghizadeh [39] et al. immobilized laccase on sodium zeolite Y (NaY) and its modified desilicated (DSY) and dealuminated (DAY) forms, and the immobilized laccase activity remained at 83.7% after storage at 3 °C for 20 days. The reason for the improved stability may be the combined effect of various forces such as hydrogen bonding, electrostatic interaction and Cu^2+^ coordination [40]. They enable laccase to maintain the secondary structure of laccase in a high temperature environment, making laccase less prone to denaturation and inactivation.

### 2.4. DCP Removal Test

**Figure 6:**
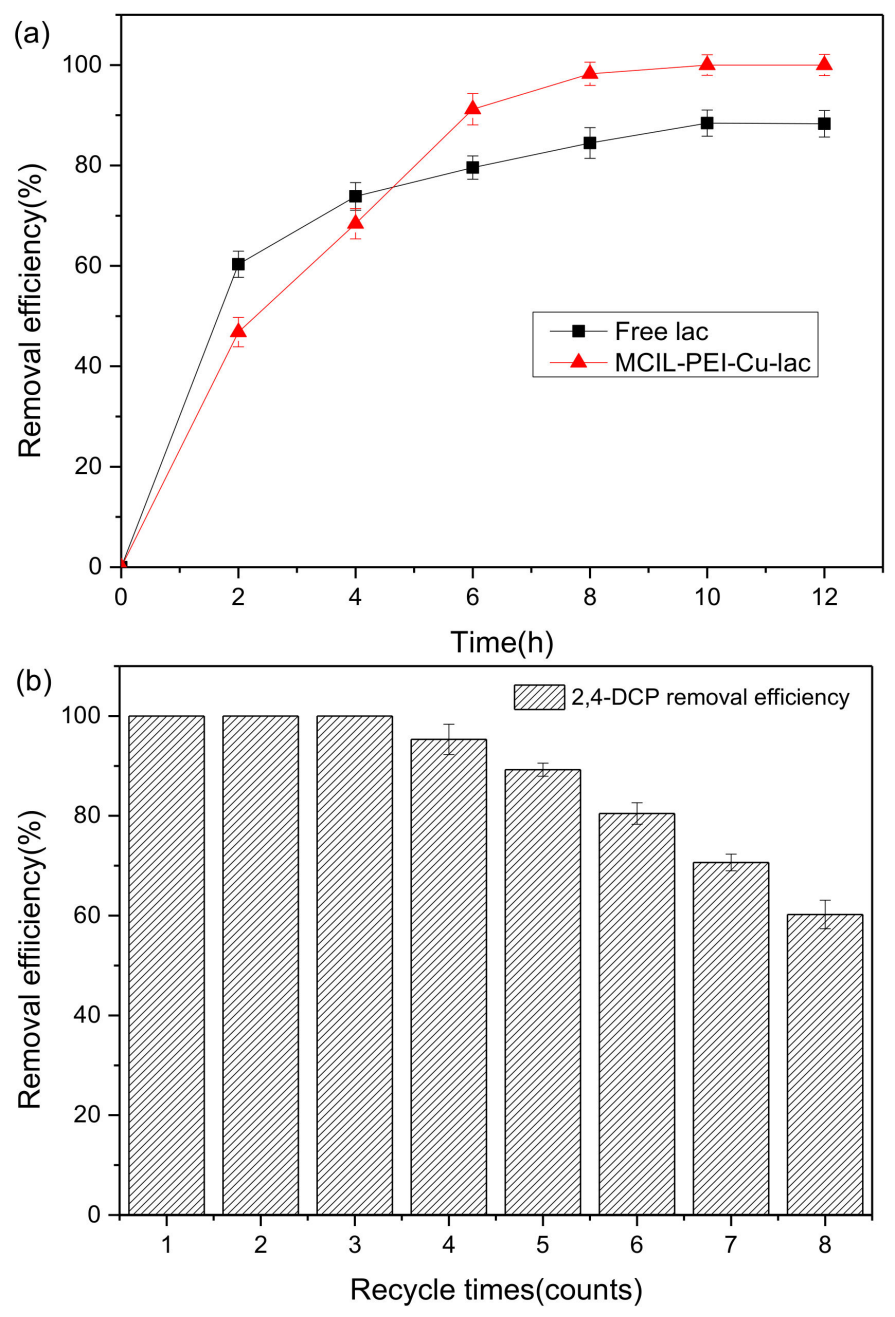
2,4-DCP removal test of various laccases (**a**). Reusability of MCIL–PEI–Cu–lac (**b**).

Figure 6a compares the removal efficiency of free laccase and MCIL–PEI–Cu–lac for 2,4-DCP (10 mg/L) in water. The maximum degradation efficiency (85.31%) of free laccase was reached at 10 h, and there was no significant improvement after a prolonged time, while MCIL–PEI–Cu–lac completely removed 2,4-DCP after 10 h. This confirms that the modification of PEI and the coordination of Cu^2+^ can not only improve the stability of the immobilized laccase MCIL–PEI–Cu–lac, but can also have excellent activity on the degradation of 2,4-DCP. The possible reason is that the abundant amino groups of PEI can form coordination bonds with laccase through the metal ion Cu^2+^. The coordination of Cu^2+^ method can reduce the deformation and desorption of laccase, and Cu^2+^ can activate laccase to a certain extent and enhance the activity of laccase [40], so that the final removal efficiency of MCIL–PEI–Cu–lac is higher than that of free laccase.

### 2.5. Reusability Test

Reusability has important value in practical applications. Compared with other materials, the magnetic material can be easily separated from the solution with a magnet. As shown in Figure 6b, MCIL–PEI–Cu–lac completely degraded 2,4-DCP (10 mg/L) in the first three cycles. After 8 cycles, the removal rate of 2,4-DCP can still reach 60.21%. On the one hand, the reason may be that the polymers generated by the degradation of phenolic pollutants will adhere to the outer surface of the carrier, occupying the active sites of laccase and reducing the oxidative degradation ability of the immobilized laccase [6]. On the other hand, the reason may be that the laccase is exposed to room temperature for too long during use, resulting in a certain decrease in the activity of the laccase [41]. At the same time, MCIL–PEI-Cu has better repeatability compared with other literature. Yang [42] et al. immobilized laccase on caged mesoporous SiO_2_ wrapped with chitosan/alginate microcapsule membrane. After 6 times of use, immobilized laccase could only remove about 50% of 2,4-DCP pollutant. Erol Alver [43] et al. immobilized laccase onto metal-chelated copolymer NPs retained about 60.0% of initial activity after 6 reaction cycles.

### 2.6. Expanding Applications Test

**Figure 7:**
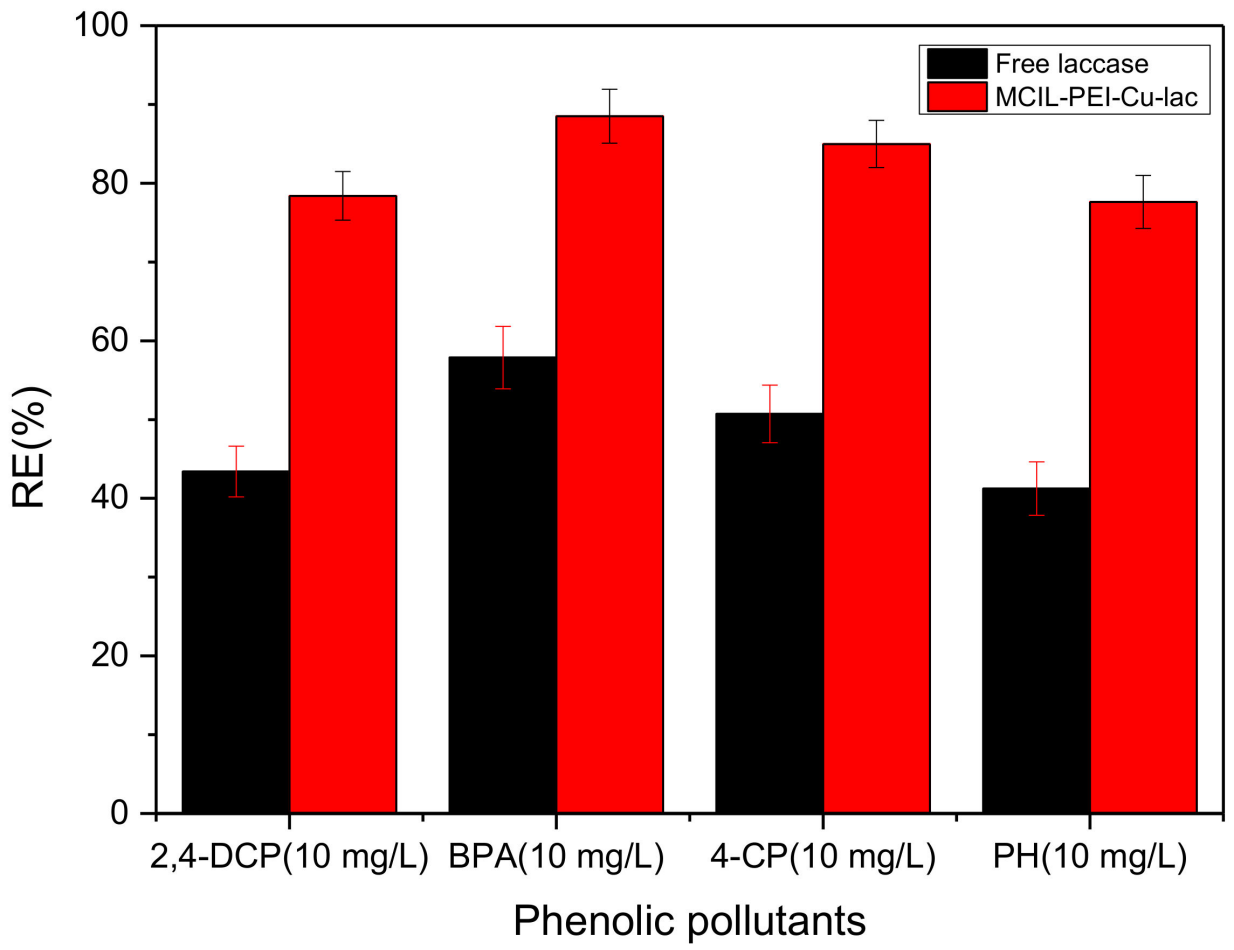
Removal efficiency of other phenolic pollutants by free laccase and MCIL–PEI–Cu–lac.

In order to further study the removal ability of immobilized laccase for other phenolic pollutants, the removal effects of immobilized laccase on 2,4-dichlorophenol (50 mg/L), bisphenol A, 4-chlorophenol and phenol (all 10 mg/L) were investigated (Figure 7). The results showed that when the removal concentration reached the 2,4-dcp concentration of 50 mg/L, the immobilized laccase still had a removal efficiency of 78.39% within 24 h. At the same time, when dealing with other phenolic pollutants, the removal effect of bisphenol A is the best, which can reach 88.51%. The treatment effect of 4-CP and phenol can also reach about 80%, which is also greatly improved compared with free laccase. Lin [44] et al. immobilized laccase onto Cu(II)- and Mn(II)-chelated magnetic microspheres and successfully applied to remove bisphenol A from water, and the maximum removal efficiency was 85.0%. Mohammadi [45] et al. immobilized laccase onto epoxy-functionalized silica particles and eventually removed 60% of 4-CP.

## 3. Materials and Methods

### 3.1. Materials

Laccase from Aspergillus oryzae was purchased by SUNSON (Ningxia, China). FeCl_3_·6H_2_O, FeCl_2_·4H_2_O, NH_3_·H_2_O (28%), 4-chlorophenol (4-CP), bisphenol A, 2,4-dichlorophenol (2,4-DCP) and phenol were provided by Aladdin (Shanghai, China). In addition, 3-chloropropyltrimethylsilane (CPTMO), 2,2-Azino-bis (3-ethylbenzothiazoline-6-sulfonic acid) (ABTS) and NaBF_4_ were supported by China National Pharmaceutical Group Corporation (Beijing, China). Chloroacetic acid and polyethylene imine (PEI) were purchased by Energy Chemical (Shanghai, China).

### 3.2. Characterizations

XRD analysis was performed on a Bruker D8 Advance instrument (Germany). FT-IR spectroscopy was obtained by a Bruker Vertex 70 FT-IR spectrometer in the range from 400 to 4000 cm^−1^ (Germany). VSM magnetic hysteresis loops were obtained on an MPMS XL-7 vibrating sample magnetometer. TGA was obtained on a Netzsch TG-209-F3 Nevio (Germany). SEM was conducted on a ZEISS Genimi500 instrument (Germany).

### 3.3. Preparation of Fe_3_O_4_ Magnetic Nanoparticles

The preparation of magnetic nano Fe_3_O_4_ refers to the previous literature [46] and is slightly modified. Firstly, 5.11 g FeCl_3_·6H_2_O and 1.99 g FeCl_2_·4H_2_O were dissolved into 100 mL deionized water and reacted at 70 °C under N_2_ protection. Then, the pH of the system was adjusted with 28% ammonia until the solution turned black completely, and reacted at 80 °C for 1.5 h. Finally, Fe_3_O_4_ particles generated by the reaction were collected and washed with deionized water until neutral. The final product was dried in a vacuum drying oven for 12 h.

### 3.4. Modification of Magnetic Fe_3_O_4_ Nanoparticles by Carboxylated Ionic Liquid

The magnetic Fe_3_O_4_ nanoparticles modified by carboxylated ionic liquids were prepared with reference to previous literature reports, and some modifications were made [24]. First, 3.0 g Fe_3_O_4_ nanoparticles were dispersed in toluene, 15 mmol 3-chloropropyltrimethylsilane (CPTMO) was added dropwise, reacted at 95 °C for 7 h under nitrogen protection, washed with ethanol and dried. After that, the obtained solid was dispersed with imidazole (15 mmol) in chloroform. After reacting at 35 °C under the protection of nitrogen for 24 h, the solid was separated by a magnet, washed with ethanol and dried. The intermediate product was then alkylated with 15 mmol of chloroacetic acid in acetonitrile at 50 °C for 12 h, separated by a magnet, washed with ethanol and dried. Finally, the intermediate product was anion-exchanged with NaBF_4_ in acetone for 48 h, separated by a magnet and washed with ethanol. The final product was dried in a vacuum oven for 24 h and designated as MCIL.

### 3.5. Preparation of Magnetic Polyethyleneimine Nanoparticles

First, 0.1 mol EDC, 0.1 mol NHS and 1.0 g MCIL were dispersed in 50 mL of citrate buffer solution (pH 7.0) and stirred at room temperature for 2 h. Then, 30 mL of PEI solution (2.5% *w*/*v*) was added, reacted at room temperature for 12 h, separated by magnet and washed with water and ethanol several times. The final product was dried in a vacuum oven for 12 h and designated as MCIL–PEI.

At the same time, in order to verify the effect of the incorporation of ionic liquid on the immobilization ability of the carrier, a comparative carrier was designed. The steps are as follows: 3.0 g Fe_3_O_4_ nanoparticles were dispersed in toluene, 15 mmol 3-chloropropyltrimethylsilane (CPTMO) was added dropwise, reacted at 95 °C for 7 h under nitrogen protection, separated by a magnet, washed several times with ethanol and dried. Afterwards, the intermediate product and 30 mL of PEI solution (2.5% *w*/*v*) were reacted at room temperature for 12 h, separated by a magnet and washed with ethanol several times. The final product was dried in a vacuum oven for 24 h and designated as MPEI.

Referring to the method reported in the previous literature [37], we introduced Cu^2+^ on MCIL–PEI. Then, 1.0 g of MCIL–PEI was dispersed in 25 mL of acetonitrile solution and 15 mmol of CuCl_2_ were added. The reaction was carried out under N_2_ protection for 48 h, and the solid was separated with a magnet and washed with deionized water. The product was designated as MCIL–PEI–Cu.

### 3.6. Laccase Activity Assay

Laccase activity was determined by measuring the rate of oxidation of ABTS by free and immobilized laccase [37,47]. An appropriate amount of laccase was added to 5 mL (1 mM) of citrate buffer at different pH, and the reaction was carried out at different temperatures for 5 min. The absorbance of the supernatant was measured by UV–vis spectrophotometer at 420 nm. One unit of laccase activity (1 U) was defined as the amount of laccase required to catalyze the oxidation of 1 μmol of ABTS in one minute under certain conditions. Each test was performed three times, and the average taken. The laccase activity was calculated according to the following formula:

Expressed activity (U/g biocatalyst) = *A* × 10^6^ × *V_t_*/(36000 × *t* × *m*_1_) (1)

Specific activity (U/g protein) = *A* × 10^6^ × *V_t_*/(36000 × *t* × *m*_2_) (2)

*A*: absorbance at 420 nm; *V_t_*: total volume (L); 36,000: molar extinction coefficient of ABTS^·+^ (M^−1^ cm^−1^); *t*: reaction time (min); *m*_1_: quality of immobilized laccase (g); *m*_2_: protein quality of immobilized laccase (g).

### 3.7. Laccase Immobilization

The optimization of enzyme immobilization process conditions is an important factor in the preparation of enzyme immobilization [48]. Enzyme loading and activity retention are important parameters for immobilized enzymes. We investigated the immobilization effect of MPEI, MCIL–PEI and MCIL–PEI–Cu under different conditions of enzyme concentration, immobilization time, pH and temperature (Figures S1–S4). Furthermore, the laccase immobilization process was optimized using protein loading and activity retention as indicators.

Laccase was immobilized on the three carriers MPEI, MCIL–PEI and MCIL–PEI–Cu under their respective optimum conditions, and the immobilized laccases were named MPEI–lac, MCIL–PEI–lac and MCIL–PEI–Cu–lac. Immobilized laccases were collected with a magnet and stored lyophilized. The determination of protein content in the supernatant was performed by the Bradford [49] method, each result performed three times, and the average taken.

### 3.8. Stability Test

Stability is an important indicator to evaluate the industrial application of laccase [50]. During the determination of thermal stability, the free and immobilized laccases were placed at 60 °C for some time, and the laccase activities were measured every hour, defining the initial laccase activity to 100%.

At the same time, in order to determine the storage stability, various laccases were stored at 3 °C for a month. Laccase activity was measured every 5 days, defining the initial laccase activity to 100%.

### 3.9. Phenolics Removal Test

In order to study the removal effect of free laccase and immobilized laccase on 2,4-DCP (10 mg/L), an appropriate amount (1.5 U) of free laccase and immobilized laccase was added to 10 mL 2,4-DCP aqueous solution, respectively, and reacted at room temperature at 12 h. The liquid chromatography measurement conditions are as follows: the mobile phase, wavelength and flow rate were 70% methanol in water, 220 nm and 1 mL/min for 10 min, respectively. Each result was measured in triplicate and averaged. The 2,4-DCP removal efficiency formula is as follows:

RE (%) = (*A*_0_ − *A_t_*)/*A*_0_ × 100 (3)

*A_0_*: corresponding peak area before removal; *A_t_*: corresponding peak area after removal.

In addition, the removal efficiency of MCIL–PEI–Cu–lac for other phenolic pollutants 2,4-DCP (50 mg/L), bisphenol A (10 mg/L), 4-chlorophenol (10 mg/L) and phenol (10 mg/L) was also determined, and the reaction was carried out at room temperature for 24 h. Bisphenol A measurement conditions: the mobile phase, wavelength and flow rate were 70% methanol in water, 290 nm and 1 mL/min for 10 min, respectively. The 4-chlorophenol and phenol measurement conditions were: the mobile phase, wavelength and flow rate were 70% methanol in water, 220 nm and 1 mL/min for 10 min, respectively.

### 3.10. Reusability Test

To determine the reusability of immobilized laccase, 1.5 U of immobilized laccase was treated with 10 mL 2,4-DCP (10 mg/L) in water at room temperature for 10 h. Afterwards, the immobilized laccase was collected with a magnet and washed three times with deionized water. The above experimental procedure was repeated after lyophilization to test the reusability.

## 4. Conclusions

In this study, polyethyleneimine PEI was combined with magnetic nanoparticles through carboxyl-functionalized ionic liquid as a bridge agent. Then laccase was immobilized on the carrier through the coordination with Cu^2+^, which has an activating effect on laccase. The results have shown that the enzyme loading and activity retention rate of MCIL–PEI–Cu–lac could reach 52.19 mg/g and 91.65%, respectively. At the same time, it has excellent thermal stability and storage stability. The laccase activity remained at 51.45% after storage at 60 °C for 6 h, and 81.13% after storage at 3 °C for 1 month. The removal rate of 2,4-DCP (10 mg/L) by MCIL–PEI–Cu–lac can reach 100%, and the removal rate remains at 60.21% after repeated use for 8 times. In addition, MCIL–PEI–Cu–lac also had a good removal effect on bisphenol A, phenol, and 4-chlorophenol. This biocatalyst has good application value in removing phenolic pollutants, and it is beneficial to the further application of the immobilized laccase in the treatment of phenol-containing wastewater.

## Figures and Tables

**Table 1:** Results of laccase immobilization ^a^.

Carriers	Laccase Immobilization		Laccase Activity Assay		
	Immobilization Efficiency (%)	Enzyme Loading (mg g^−1^)	Expressed Activity (U g^−1^ Biocatalyst)	Specific Activity (U g^−1^ Protein)	Activity Retention (%)
MPEI	32.04 ± 0.07	30.21 ± 0.08	42.76 ± 0.15	1415.36 ± 3.48	47.69 ± 0.14
MCIL–PEI	36.26 ± 0.11	34.17 ± 0.09	58.67 ± 0.13	1717.01 ± 3.14	57.85 ± 0.12
MCIL–PEI–Cu	55.34 ± 0.13	52.19 ± 0.12	141.97 ± 0.16	2720.03 ± 3.65	91.65 ± 0.16

^a^ The activity of free laccase is 2967.84 U g^−1^ protein.

## Data Availability

Not applicable.

## Supplementary Materials

The following supporting information can be downloaded at: https://www.mdpi.com/article/10.3390/molecules27238522/s1, Figure S1: Effect of time on laccase immobilization; Figure S2: Effect of enzyme concentration on laccase immobilization; Figure S3: Effect of pH on laccase immobilization; Figure S4: Effect of temperature on laccase immobilization.
