# Supplementary material for: Magnetic Polyethyleneimine Nanoparticles Fabricated via Ionic Liquid as Bridging Agents for Laccase Immobilization and Its Application in Phenolic Pollutants Removal
Source: Molecules. 2022 Dec 3;27(23):8522. doi: 10.3390/molecules27238522 (PMC9738685; doi:10.3390/molecules27238522)
Supplement: Supplementary file 1 [file molecules-27-08522-s001.zip › molecules-2071710-supplementary.pdf]

Supporting information

1 Methods

1.1 Time

The carrier (MCIL, MCIL-PEI or MCIL-PEI-Cu, 15 mg) was sonicated into 5 mL citrate buffer solution (0.1 M, pH 3.0), and then 5 mL laccase solution was added in the suspension. The system was shaken at 30°C for 7 h. Measure the protein concentration of the supernatant every hour. Bradford method was used to determine the protein content to calculate the loading of immobilized laccase. After the time reached 5 h, the protein loading did not increase significantly, and the optimal immobilization time was 5 h.

1.2 Enzyme concentration

The carrier (MCIL, MCIL-PEI or MCIL-PEI-Cu, 15 mg) was sonicated into 10 mL mixture containing citrate buffer solution (0.1 M, pH 3.5) and then laccase solution (concentration: 0.3-0.7 mL Laccase solution/mL). The system was shaken at respective optimum temperature conditions for 6 h. The immobilized enzyme was separated with a magnet and washed several times with the buffer solution. The activity of immobilized laccase was examined and the maximum laccase activity was defined as 100%. The optimal immobilization concentration of each carrier was 0.5 mL enzyme solution/mL for MPEI, 0.5 mL enzyme solution/mL for MCIL-PEI, and 0.6 mL enzyme solution/mL for MCIL-PEI-Cu.

1.3 pH

The carrier (MCIL, MCIL-PEI or MCIL-PEI-Cu, 15 mg) was sonicated into 10 mL laccase solution (pH 3.0-5.0). The system was shaken at 30°C for 6 h. The immobilized enzyme was separated with a magnet and washed several times with the buffer solution. The activity of immobilized laccase was examined and the maximum laccase activity was defined as 100%.

1.4 Temperature

The carrier (MCIL, MCIL-PEI or MCIL-PEI-Cu, 15 mg) was sonicated into 10 mL laccase solution (pH 3.5, 3.5 or 4.0). The system was shaken at 20-50°C for 6 h. The immobilized enzyme was separated with a magnet and washed several times with the buffer solution. The activity of immobilized laccase was examined and the maximum laccase activity was defined as 100%.

2 Results

2.1 Time

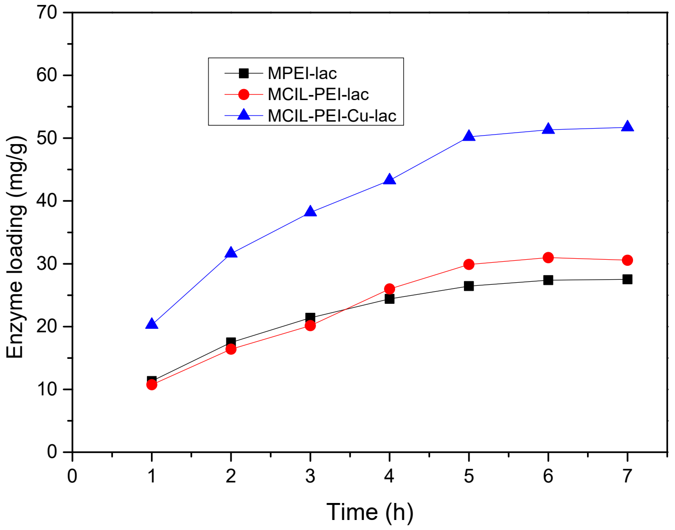

Figure S1. Effect of time on laccase immobilization

2.2 Enzyme concentration

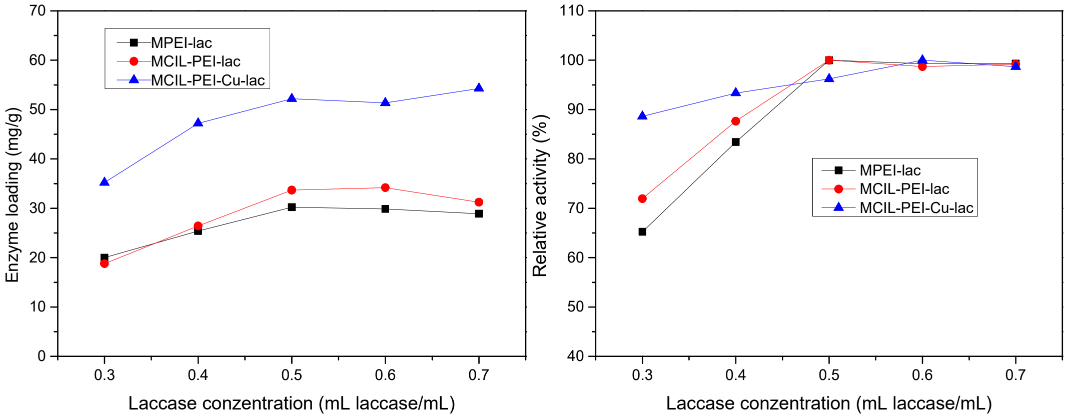

Figure S2. Effect of enzyme concentration on laccase immobilization

2.3 pH

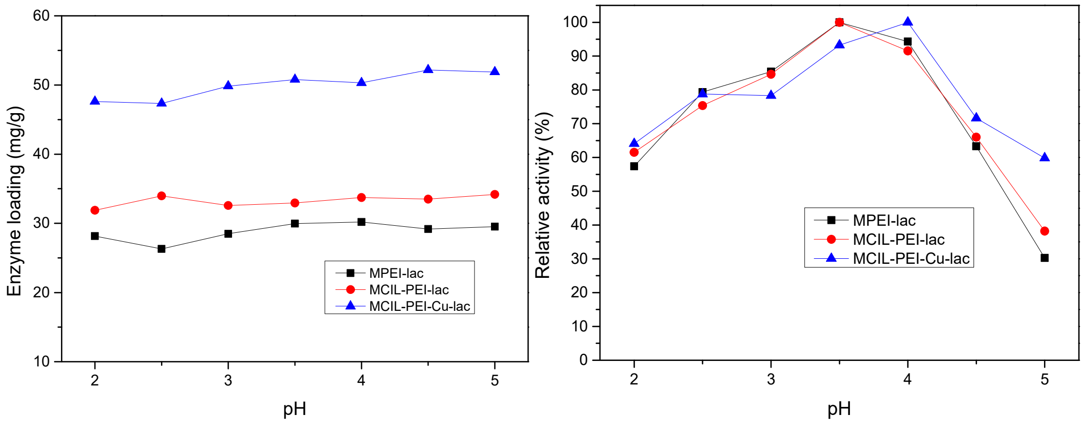

Figure S3. Effect of pH on laccase immobilization

2.4 Temperature

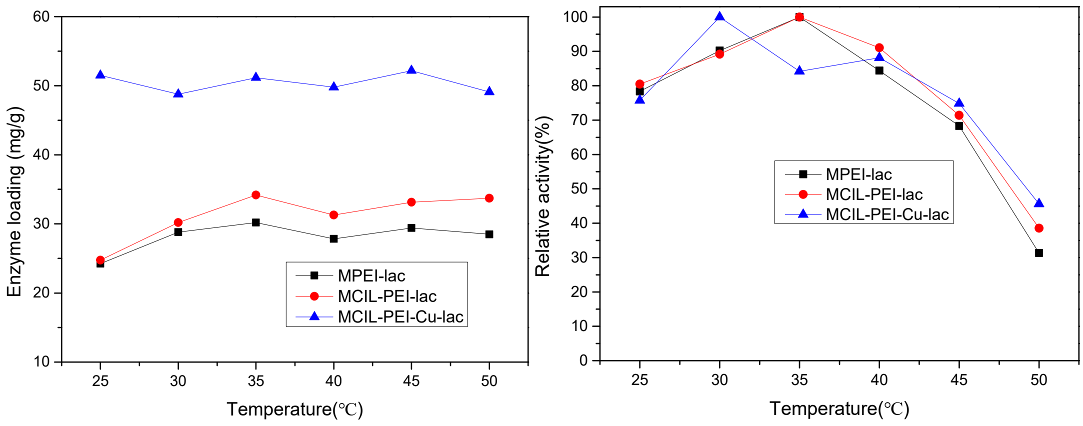

Figure S4. Effect of temperature on laccase immobilization
